# Supplementary material for: Bringing SEM and MSI Closer Than Ever Before: Visualizing Aspergillus and Pseudomonas Infection in the Rat Lungs
Source: J Fungi (Basel). 2020 Oct 30;6(4):257. doi: 10.3390/jof6040257 (PMC7711807; doi:10.3390/jof6040257)
Supplement: Supplementary file 1 [file jof-06-00257-s001.pdf]

Supplementary Materials: Bringing SEM and MSI Closer Than Ever Before: Visualizing *Aspergillus* and *Pseudomonas* Infection in the Rat Lungs

Tereza Juříková [1,2], Dominika Luptáková [1], Olga Kofroňová [1], Anton Škríba [1], Jiří Novák [1], Helena Marešová [1], Andrea Palyzová [1], Miloš Petřík [3], Vladimír Havlíček [1,4*] and Oldřich Benada [1,5*]

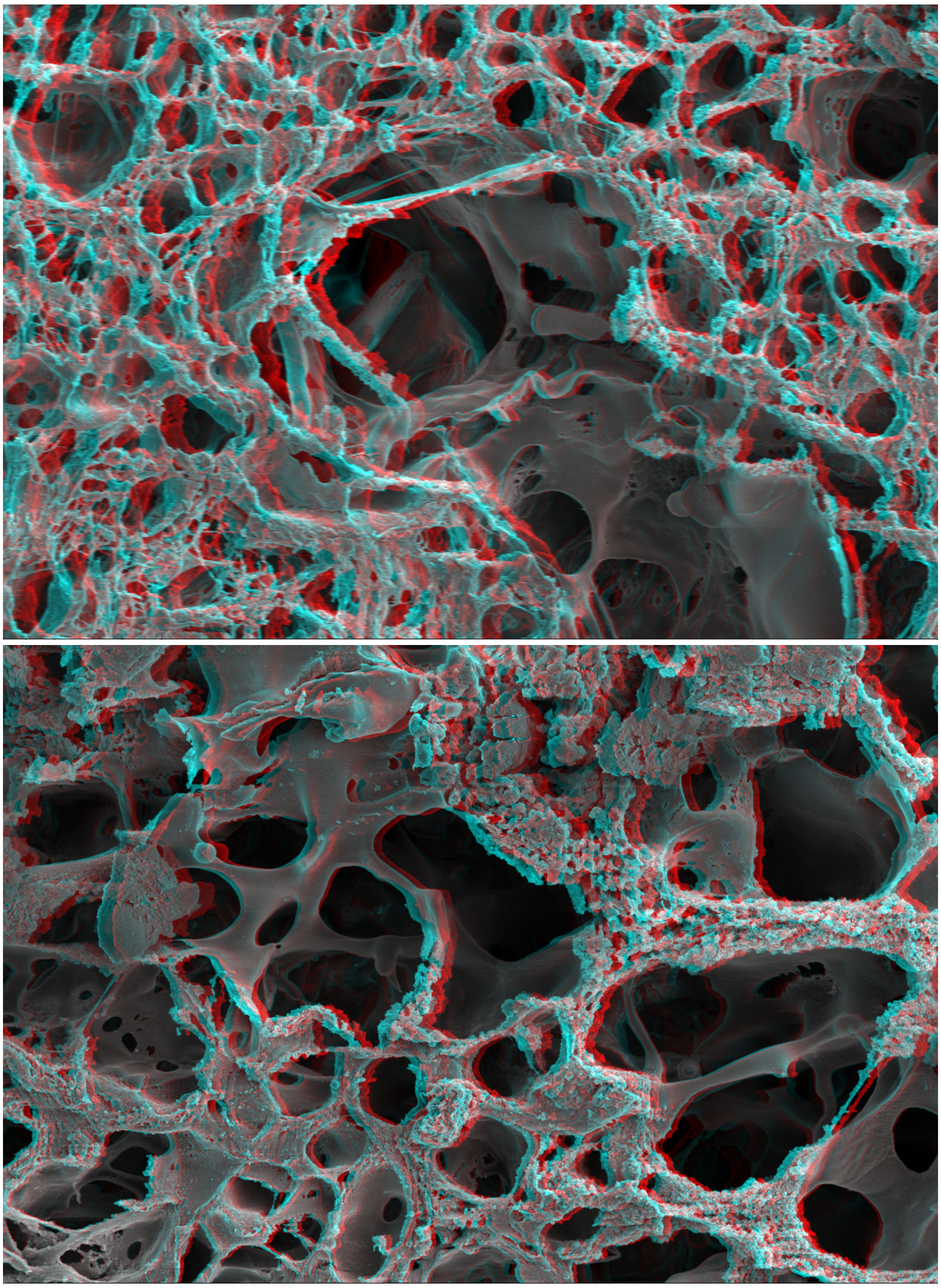

Figure S1. Stereo images (R-GB anaglyphs) of float-fixed cryosection from rat lung tissue infected with *A. fumigatus* taken from different parts of the section at a primary magnification of 5000× (upper panel, approximate field of view (FOV): 82.9 µm × 55.3 µm) and 8000× (lower panel, approximate FOV: 51.8 µm × 34.5 µm). The stereo pair tilt angle difference is 6°; red-cyan or red-green glasses are required for proper viewing. Instead of scale bars, the FOV size is given for both anaglyphs.

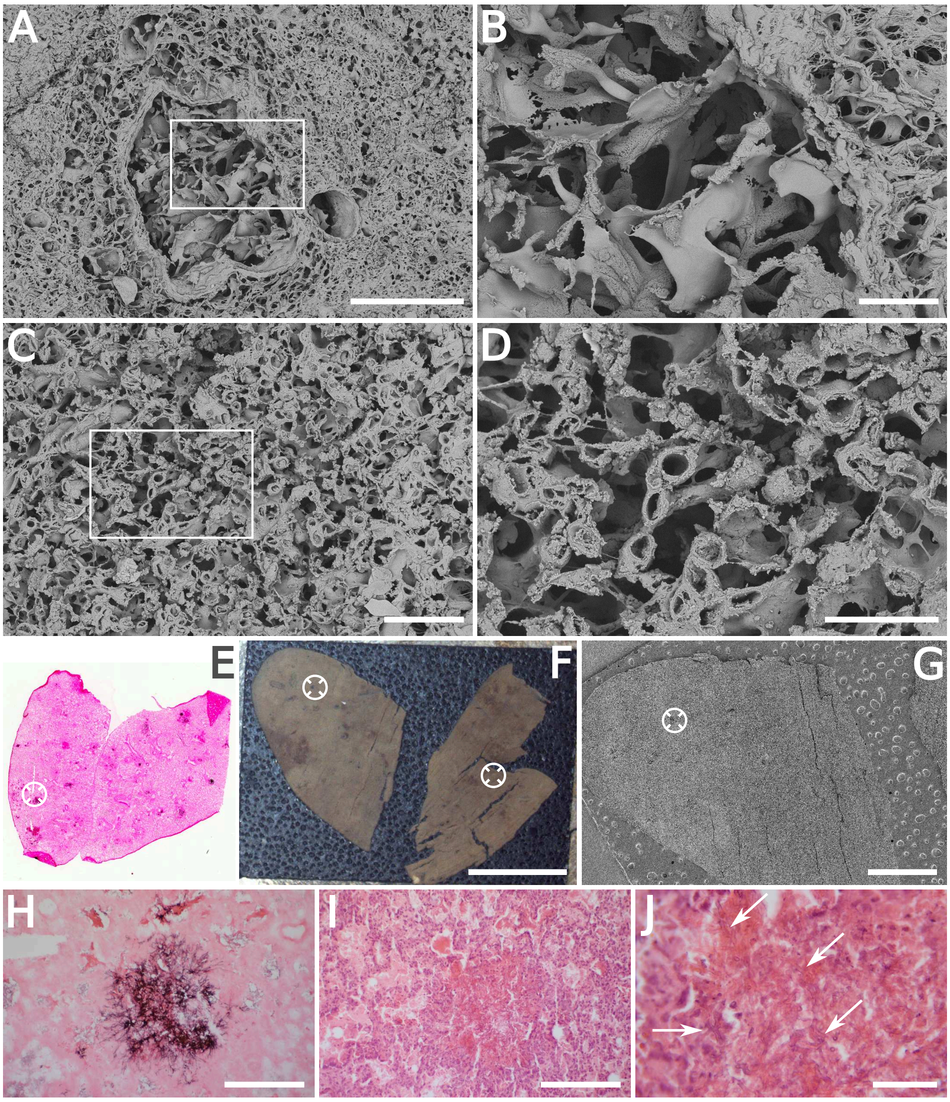

Figure S2. A comparison of scanning electron microscopy and histology of *Aspergillus* fumigatus infected rat lung tissue. Panels A, B, C, D correspond to the same panels as of Figure 3 in the main text. Proper locations of areas from which SEM images were taken are marked in the whole lobe section's images (panels E, F, and G). The panels F and G's marked positions were accurately set by scanning electron microscope (SEM) navigation system. The position marked in panel E is approximate. Panel E shows optically scanned Grocott's methenamine silver-stained (GMS) consecutive section. Panel F shows a vacuum dried section on SEM sample mount, and panel G represents the SEM navigation image of the left section shown on the sample mount in panel F. The lowest panel row presents Grocott's methenamine silver-stained consecutive section (panel H) and hematoxylin-eosin (H&E) stained consecutive section (panels I and J). The location of panels H, I, and J should correspond to the marked area in the panels E, F, and G. The *Aspergillus* hyphae are barely visible in the H&E stained section when compared with GMS staining. Scale bars: 100 µm (A); 20 µm (B, C); 10 µm (D); 5 mm (F); 2 mm (G); 250 µm (H, I); 50 µm (J).

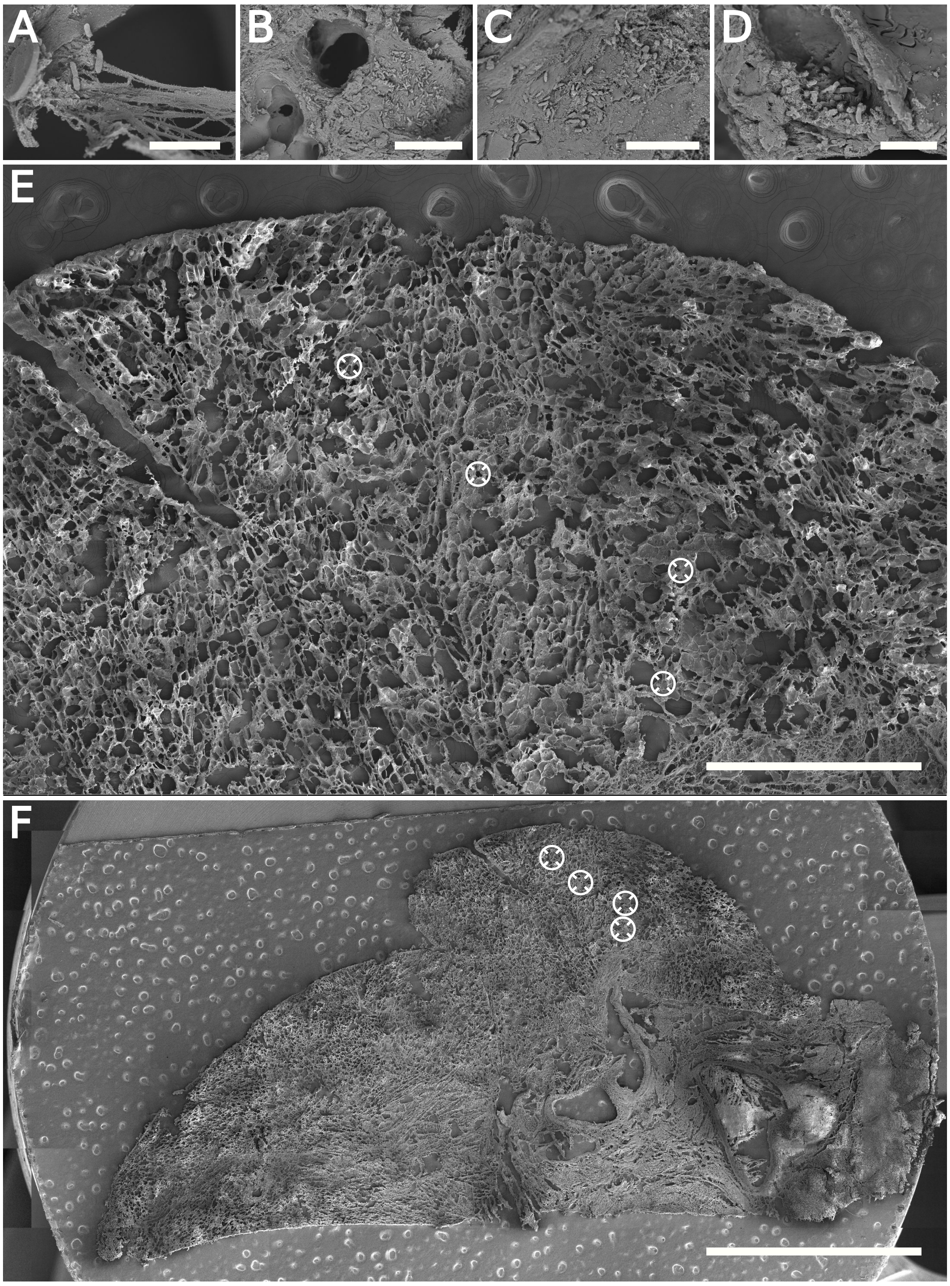

Figure S3. Scanning electron microscopy of *Pseudomonas aeruginosa* infected rat lung tissue and exact localization of the areas from which high-resolution images were taken. Panels in the upper row correspond to the panel H, E, F, and G of Figure 3 in the main text. Their exact locations in the lobe section are marked in the same order in the low-resolution panels below. The middle image (panel E) corresponds to the panel E of Figure 4 in the main text. The lower panel F represents the navigation image mapping of the whole lobe section. The panels E and F's marked positions were accurately set by scanning electron microscope navigation system. Scale bars: 5 µm (A); 10 µm (B); 20 µm (C); 5 µm (D); 1 mm (E); 5 mm (F).

1. Materials and Methods

1.1. Anaglyph construction

R-GB anaglyphs construction is specific for the type of SEM device. In FEI Nova NanoSEM 450 the sample must be at an eucentric (analytical) position. Scan rotation was adjusted to -90° or +90°. This set up the tilt axis perpendicular to the horizontal image axis. A stereo pair of the area of interest

was recorded with tilt difference of 6° (usually -6° and 0°). The anaglyph was constructed declaring -6° tilted image (8-bit) as red and zero tilted image (8-bit) as green and also as blue. Final summing of those three images in 24-bit RGB color space then produced R-GB anglyph. If the stereo pair tilt axis moves during tilting its displacement can be rectified in some software (e.g., Stereo modul of Analysis 3.2 software, EMSIS, GmbH, Muenster, Germany).

1.2. A comparison of histology and SEM on the consecutive sections

Consecutive sections from deep-frozen rat lung tissue infected by *A. fumigatus* were individually processed by float-fixation method for SEM and by Grocott's methenamine silver staining (GMS) or hematoxylin-eosin staining (H&E) for optical microscopy. Float-fixation and GMS procedures are described in the main text, in the *Material and Methods* section.

H&E staining were performed as follows. Dried, thaw-mounted, 15 mm-thick frozen sections (Leica Microsystems GmbH, Wetzlar, Germany) were rehydrated in the ethanol series (100, 95, and 70%), washed in distilled water and stained by hematoxylin (Sigma-Aldrich, Czech Republic) and eosin Y (VWR Chemicals, Czech Republic). Dehydrated sections (ethanol, 95, and 100%) were cleared in xylene and mounted in DPX medium (Sigma-Aldrich, Czech Republic). Final mounts were examined under a DN45 light microscope (LAMBDA PRAHA Ltd., Prague, Czech Republic) equipped with a Canon EOS 700D Digital SLR camera.

1.3. Exact assignment of the locations of the bacteria in the section

A Navigation Montage option of the SEM software (Helios NanoLab) was used to map the whole lung lobe section at low resolution. Then the pyoverdine E protonated molecule map from the consecutive section was used to settle the region of interest with possible presence of bacteria *Pseudomonas aeruginosa*. Next the sets of images with increasing magnification were recorded in this region.
